# Supplementary material for: Recombinant hirudin attenuates pulmonary hypertension and thrombosis in acute pulmonary embolism rat model
Source: PeerJ. 2024 Apr 5;12:e17039. doi: 10.7717/peerj.17039 (PMC11000639; doi:10.7717/peerj.17039)

β -actin

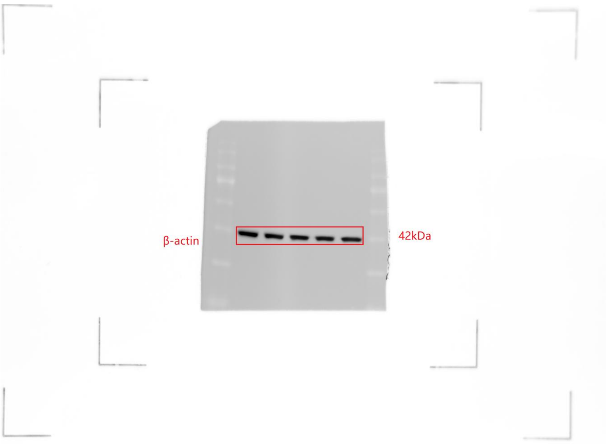

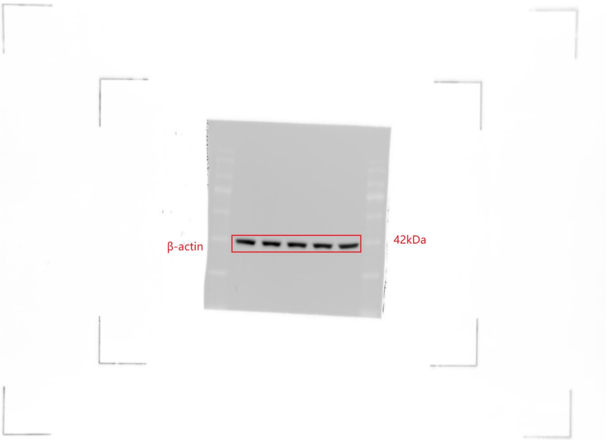

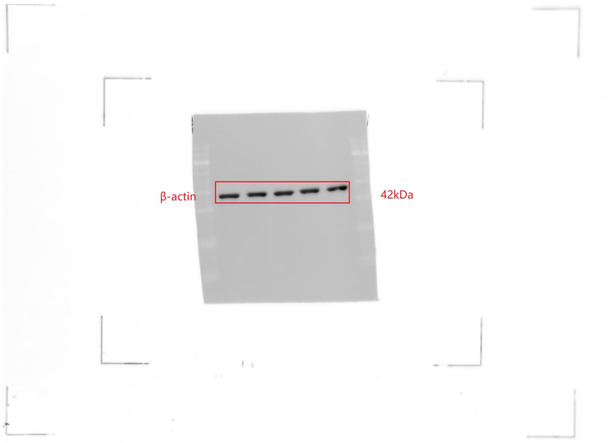

ERK

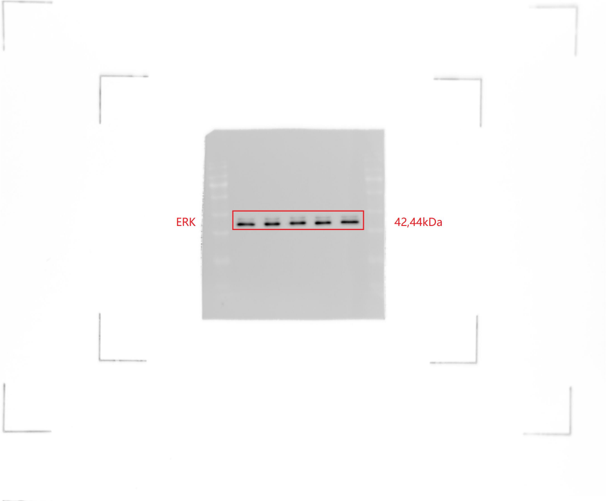

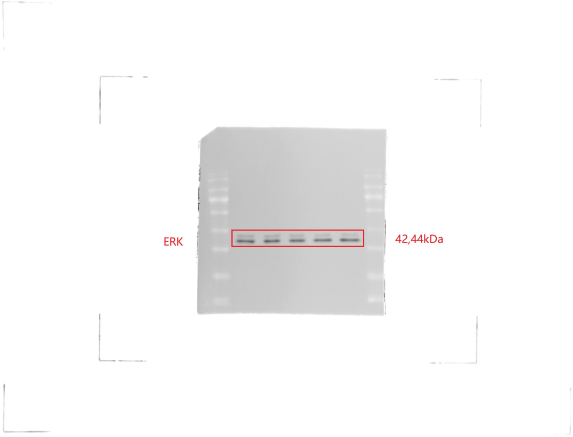

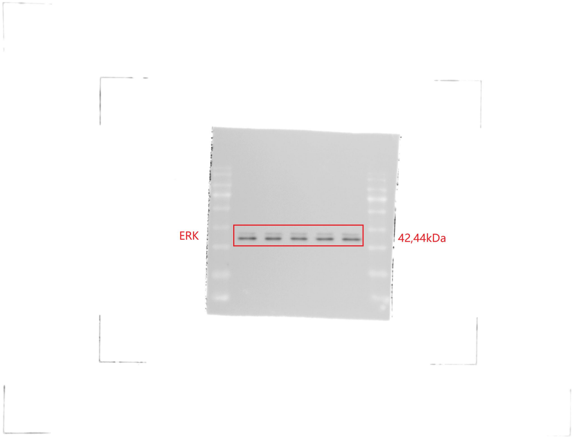

p-ERK

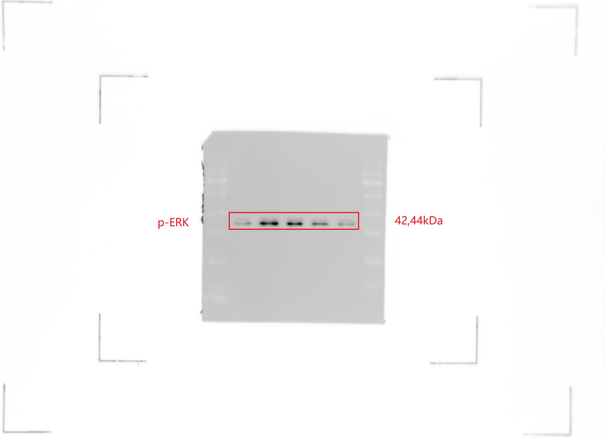

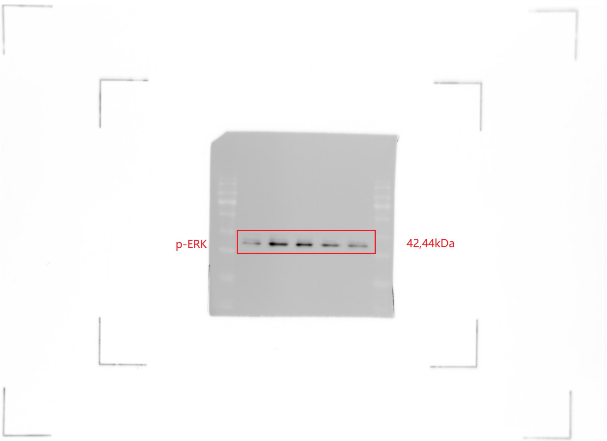

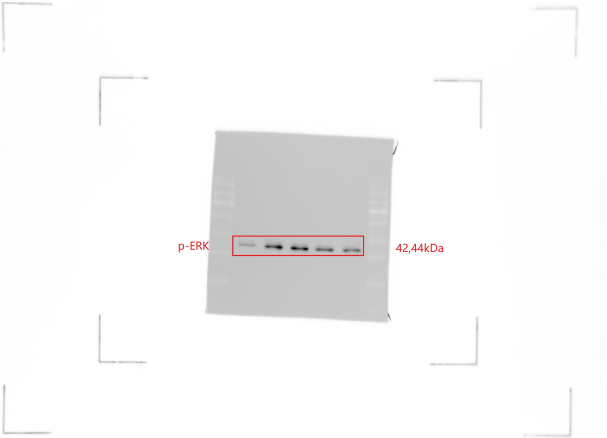

P65

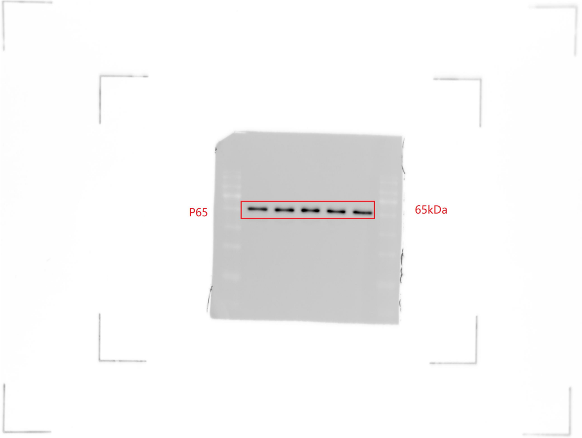

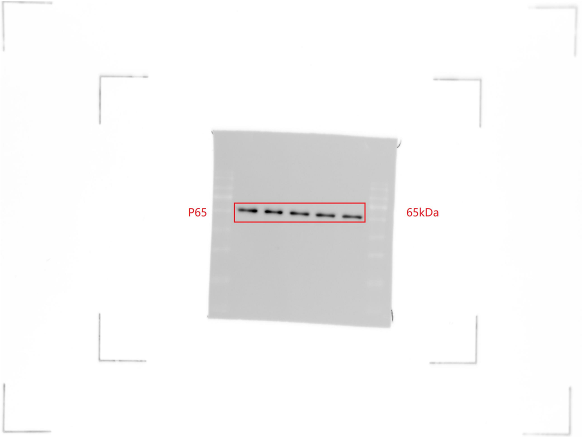

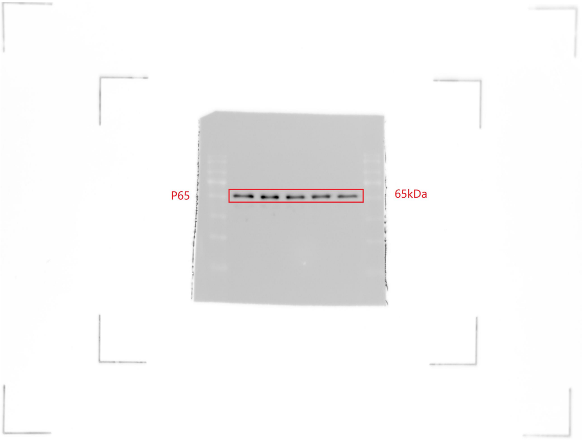

P-p65

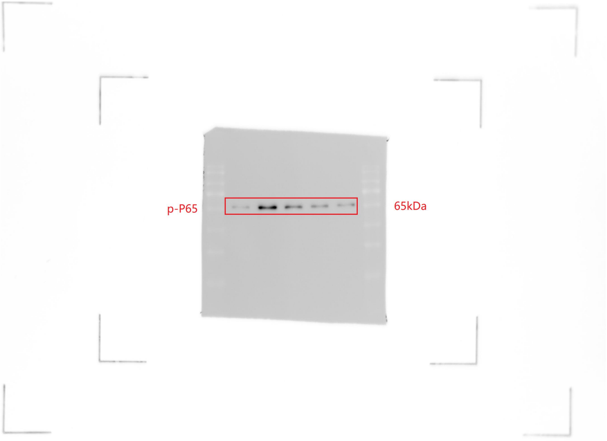

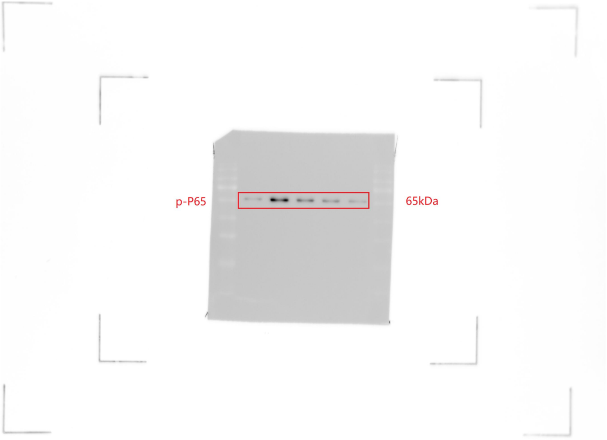

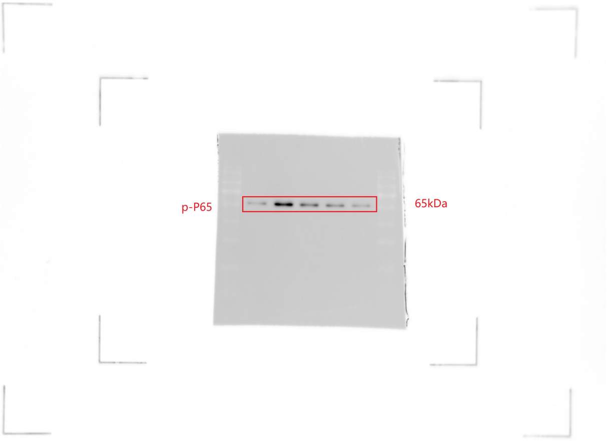

Supplement: Supplemental Information 3 [file peerj-12-17039-s003.pdf]
